# Supplementary material for: Efficient electrospray deposition of surfaces smaller than the spray plume
Source: Nat Commun. 2023 Aug 14;14:4896. doi: 10.1038/s41467-023-40638-7 (PMC10425365; doi:10.1038/s41467-023-40638-7)
Supplement: Supplementary file 1 — Supplementary Information Efficient Electrospray Deposition of Surfaces Smaller than the Spray Plume [file 41467_2023_40638_MOESM1_ESM.pdf]

Supplementary Information

Efficient Electrospray Deposition of Surfaces Smaller than the Spray Plume

Sarah H. Park[1], Lin Lei[2], Darrel D'Souza[2], Robert Zipkin[3], Emily T. DiMartini[4], Maria Atzampou[4], Emran O. Lallow[2], Jerry W. Shan[2], Jeffrey D. Zahn[4], David I. Shreiber[4], Hao Lin[2], Joel N. Maslow[5], Jonathan P. Singer[1,2*]

[1] Department of Materials Science and Engineering, Rutgers, The State University of New Jersey, Piscataway, NJ 08854, USA.

[2] Department of Mechanical and Aerospace Engineering, Rutgers, The State University of New Jersey, Piscataway, NJ 08854, USA.

[3] MedChem 101 LLC, Plymouth Meeting, PA.

[4] Department of Biomedical Engineering, Rutgers, The State University of New Jersey, Piscataway, NJ 08854, USA.

[5] GeneOne Life Science, Seoul, South Korea.

[*]Corresponding Author Email: jonathan.singer@rutgers.edu (JPS)

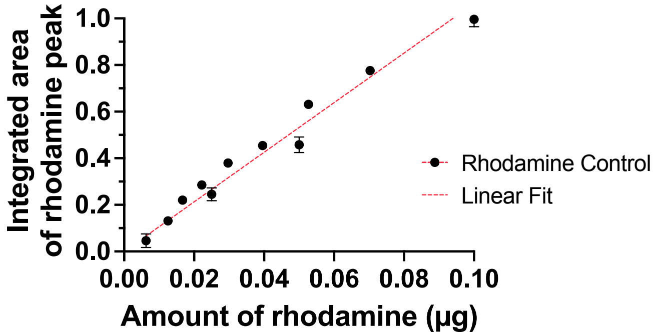

Supplementary Figure 1. Calibration curve of tracer material, rhodamine B. Known concentrations of rhodamine in water was measured using UV-vis spectrophotometry where the peak signal was integrated to obtain an area to then correlate with the known concentration. Each concentration was measured in triplicate. Error bars indicate standard deviation.

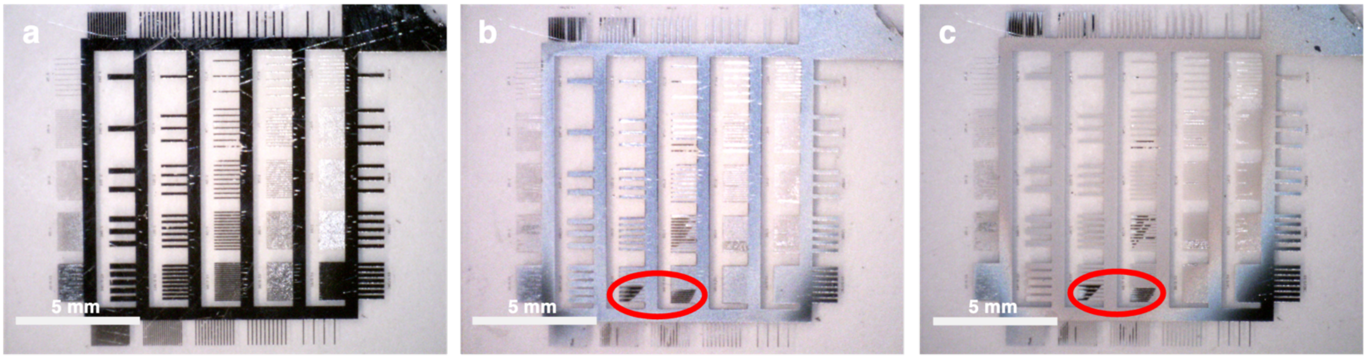

Supplementary Figure 2. Comparison of self-limiting and non-self-limiting material. Photographs of a **a** bare electrode test pattern fabricated on borosilicate glass and the test pattern sprayed with **b** trehalose and **c** PVP. Regions in **b** and **c** where the pattern is not coated is due to damage in the pattern itself, thus leading to a lack of contact grounding. Damage on the chip is outlined in red for both **b** and **c**.

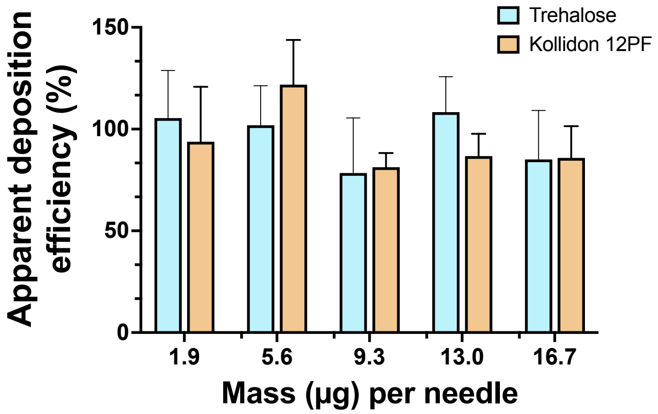

Supplementary Figure 3. Effect of dosage on deposition efficiency. Deposition efficiencies of a non-self-limiting material, trehalose, and a self-limiting material, Kollidon 12PF, at various dosages. From left to right, the dosages are for a 10-, 30-, 50-, 70-, and 90-min spray time. For each time, n = 3 per each material. Error bars indicate standard deviation.

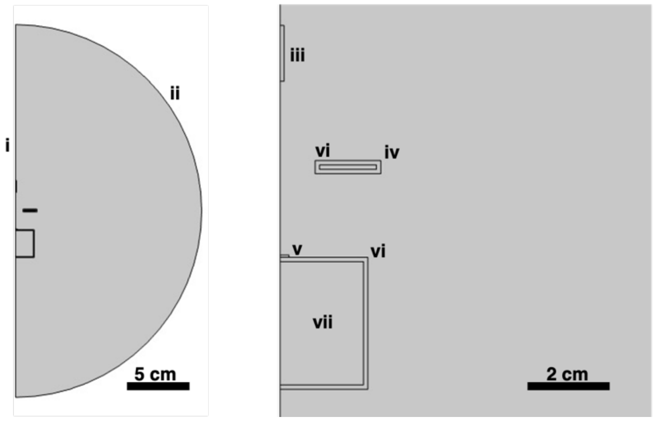

Supplementary Figure 4. Pictures of the simulation cell for spray simulation. The simulation was conducted in COMSOL 6.0 with an axisymmetric symmetry, revolved around face i. A grounded boundary (representing other targets and deionization by the environment) was placed 15 cm away (face ii). A needle domain was set to 7.4 kV (region iii) 4 cm above the target with dimensions similar to the needle. A ring domain was set to 0.91 kV (region iv) 2 cm above the target with dimensions similar to the ring. A 1 cm radius grounded domain was used as the target

(region v). An insulating layer (region vi) with surface charge was placed on this ring and on an extractor ground region (region viii). The extractor had similar dimensions to the extractor ground and was simulated as either floating or grounded. The mesh was converged visually based on the smoothness of the potential maps. For the simulation, 1,000 field lines were emitted from the bottom of region iii, and their termination was tracked.

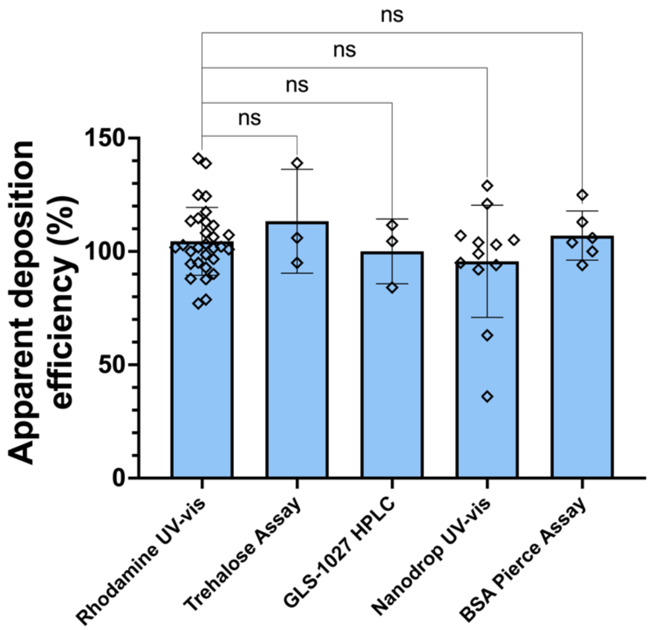

Supplementary Figure 5. Comparison of efficiency measurement methods. Apparent deposition efficiencies measured using a UV-vis approach with rhodamine B as an internal standard (n = 30) compared to other methods of measurement using trehalose assay kit (n = 3), HPLC (n = 3), Nanodrop UV-vis (n = 12), and Pierce 660 nm assay (n = 6). Pairwise comparisons were conducted using ANOVA multiple comparisons tests. Error bars indicate standard deviation. Each point indicates a single measurement.

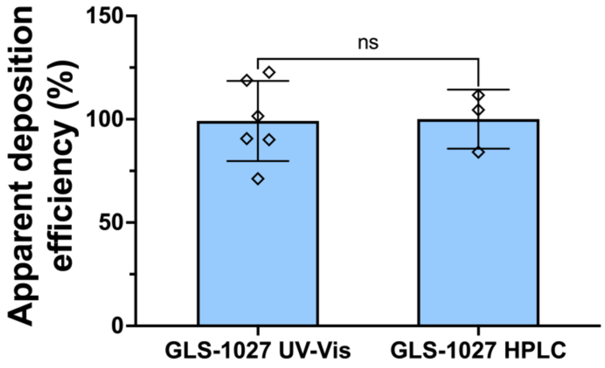

Supplementary Figure 6. Comparison of UV-vis and HPLC measurements for GLS-1027. Apparent deposition efficiencies of GLS-1027 measured via UV-vis and HPLC, reporting efficiencies of 99 ± 18% (n = 6) and 100 ± 14% (n = 3), respectively, showing no statistical significance. Pairwise comparison was conducted using an unpaired, two-tailed student t-test. Error bars indicate standard deviation. Each point indicates a single measurement.

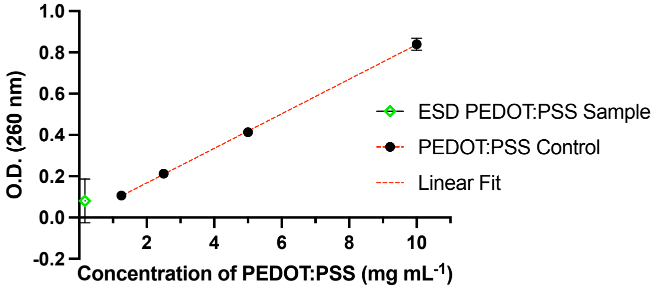

Supplementary Figure 7. Calibration curve of PEDOT:PSS. All absorbances were measured using Nanodrop (OD = 260 nm). Stock PEDOT:PSS concentrations were measured using a 1:1 serial dilution starting at 10 mg mL^{-1} until 1.25 mg mL^{-1} to generate a linear fit. Each concentration was measured in triplicate. Sprayed PEDOT:PSS samples were then overlaid on top of the calibration curve. Error bars indicate standard deviation.

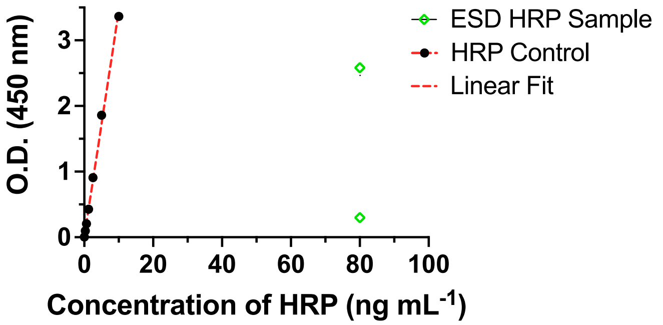

Supplementary Figure 8. Calibration curve of BSA generated from ELISA assay. Stock HRP concentrations were measured using a 1:1 serial dilution starting at 10 ng mL^{-1} until 0.3125 mg mL^{-1} to generate a linear fit. Each concentration was measured in quadruplicate. Sprayed HRP samples were then overlaid on top of the calibration curve.

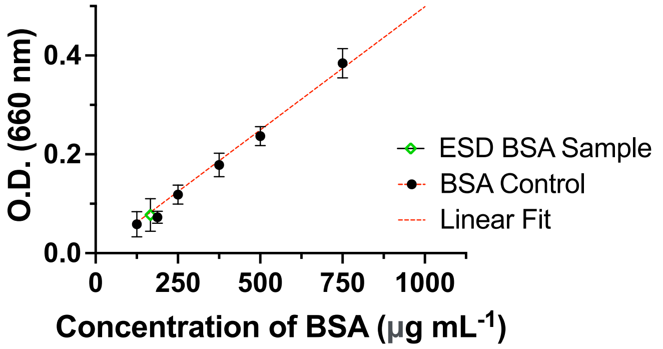

Supplementary Figure 9. Calibration curve of BSA generated from a Pierce 660 assay. Stock BSA concentrations were measured using a 1:1 serial dilution starting at 1000 μg mL^{-1} until 125 μg mL^{-1} to generate a linear fit. Each concentration was measured in triplicate. Sprayed BSA samples were then overlaid on top of the calibration curve. Error bars indicate standard deviation.

Supplementary Table 1. Experimental spray conditions of all samples.

Material	Conc.	Time (min)	Flowrate (mL/hr)	Voltage 1 (kV)	Voltage 2 (kV)	Temperature (˚C)	Spray Distance (cm)	Humidity
Trehalose (T1 - MNA)	0.2 (w/v)%	30	0.1	7.25	0.41	29.1	4	23%
				7.6		30.6		23%
				7.4		30.6		23%
				7.5		30.6		23%
				7.7		25.6		16%
				7.4		24.8		18%
PEGDA	0.2 (w/v)%	30	0.1	6.7	0.41	24.7	4	42%
				7.25		23.3		45%
				7.9		26.5		20%
				7.3		24.6		18%
				6.8		23.1		20%
				6.8		24.5		20%
PVP (Kollidon 12PF)	0.2 (w/v)%	30	0.1	6.6	0.41	25.8	4	19%
				6.9		26.1		26%
				6.7		22.7		21%
				7.2		26.8		22%
				7.8		25.7		24%
				8		24.1		24%
GLS-6150	0.05 (w/v)%	30	0.1	8	0.41	26.5	4	21%
				7.8		25.5		21%
				7.8		26.1		21%
				7.2		26.6		21%
				7.5		25.3		21%
				7.4		26.1		21%
9 to 1 Trehalose to Horseradish Peroxidase	0.2 (w/v)%	30	0.1	7.55	0.41	20.6	4	19%
				7		20.5		19%
				7.2		20.7		20%
				7.2		24.1		19%
				7.4		24.1		18%
				7.2		24		19%
GLS-1027		30	0.1	6.4	0.41	23.1	4	20%

				6.7		23.8		22%
				6.7		23.8		22%
	0.2 (w/v)%			7.6		24.9		18%
				6.8		25.1		21%
				7.6		25.1		22%
P3KT	0.1 (w/v)%	30	0.1	7.6	0.41	26.5	4	19%
				7.4		26		21%
				8.4		26.1		19%
				8.7		23.5		22%
				7.4		26.6		23%
				7.4		26.4		26%
PEDOT:PSS	0.2 (w/v)%	30	0.1	8	0.41	24.1	4	21%
				8.7		25.3		21%
				8.7		25.9		21%
				8.8		24.4		20%
				9		23.5		21%
				8.2		25.9		21%
BSA	0.2 (w/v)%	30	0.1	8.2	0.41	24.1	4	21%
				8.4		26		21%
				7.6		25.4		21%
				8		26		21%
				8.2		26.1		21%
				8.4		26.1		20%
Trehalose (T2 - Silicon wafer)	0.2 (w/v)%	30	0.1	7	0.41	24.1	4	20%
				7.8		24.1		24%
				7.4		24.1		25%
				7.3		24.1		23%
				7.2		24.3		25%
				7.2		25.2		22%
Trehalose (T3 - Borosilicate uChip)	0.2 (w/v)%	30	0.1	5.8	0.41	21	4	19%
				6.7		24.7		21%
				6		24.5		21%
				7		23.8		20%
				7.2		22.5		21%
				7.2		26.1		21%

WO1 - No Prespray	0.2 (w/v)%	30	0.1	7.1	0.41	24.1	4	21%
				7.8		24.1		22%
				7.4		24.1		23%
				7.3		24.1		17%
				7.2		24.1		17%
				6.5		24.2		17%
WO2 - No Insulating Mask	0.2 (w/v)%	30	0.1	7.2	0.41	24.6	4	18%
				7.2		24		19%
				8		25.1		18%
				8		25.1		18%
				8.6		25.1		20%
				7.5		25.2		23%
WO3 - No Insulating Environment	0.2 (w/v)%	30	0.1	5.65	0.6	25	4	19%
				5.7		24.9		20%
				5.6		24.1		21%
				5.6		25.2		22%
				5.7		25.1		22%
				5.6		25		22%
WO4 - No Grounded Extractor	0.2 (w/v)%	30	0.1	7.5	0.6	25.1	4	20%
				7.7		25.1		22%
				7.5		25.1		23%
				7.5		25		22%
				7.3		25.1		23%
				7.3		25.1		19%
WO5 - No Focus Ring	0.2 (w/v)%	30	0.1	8.2	0.41	24.7	4	17%
				8.3		25.1		17%
				8		25.1		17%
				7.9		25.1		17%
				8.2		25.1		17%
				7.6		25.1		22%
9:1 Trehalose:HRP (ELISA)	0.2 (w/v)%	30	0.1	7.7	0.41	25.1	4	22%
				7.9		25.1		23%
GLS-1027 (HPLC)	0.2 (w/v)%	90	0.1	7	0.41	29.8	4	26%
				7.2		30.1		26%
				7.6		30.1		26%

Trehalose Assay	0.2 (w/v)%	30	0.1	8	0.41	25.2	4	21%
				7.8		26.5		22%
				7.8		22.8		21%
