# Supplementary material for: Efficient electrospray deposition of surfaces smaller than the spray plume
Source: Nat Commun. 2023 Aug 14;14:4896. doi: 10.1038/s41467-023-40638-7 (PMC10425365; doi:10.1038/s41467-023-40638-7)
Supplement: Supplementary file 3 — Description of Additional Supplementary Information [file 41467_2023_40638_MOESM3_ESM.pdf]

Description of Additional Supplementary Data

File name: Supplementary Video 1.

Description: ESD using a 0.2 (w/v)% trehalose solution in 80:20 ethanol:water onto a MNA. The video is played at 20X speed.
